# Synthesis and Characterization of Bis[1]benzothieno[3,2-*b*:2′,3′-*d*]pyrroles: Quantitative Effects of Benzannulation on Dithieno[3,2-*b*:2′,3′-*d*]pyrroles

**DOI:** 10.3390/molecules23092279

**Published:** 2018-09-06

**Authors:** Rylan M. W. Wolfe, Evan W. Culver, Seth C. Rasmussen

**Affiliations:** Department of Chemistry and Biochemistry, North Dakota State University, NDSU Dept. 2735, P.O. Box 6050, Fargo, ND 58108-6050, USA; rylanwolfe@gmail.com (R.M.W.W.); evan.wayne.culver@ndsu.edu (E.W.C.)

**Keywords:** fused-ring thiophenes, heteroacenes, dithieno[3,2-*b*:2′,3′-*d*]pyrroles, benzannulation, structure-function relationships

## Abstract

The synthesis of four *N*-functionalized bis[1]benzothieno[3,2-*b*:2′,3′-*d*]pyrroles (BBTPs) is reported in order to provide a more detailed characterization of these fused-ring units, as well as increase the scope of known BBTP units available for application to conjugated materials. The optical, electronic, and structural properties of the resulting BBTP units have been compared to the parent *N*-alkyl- and *N*-aryl-dithieno[3,2-*b*:2′,3′-*d*]pyrroles (DTPs), as well as their corresponding 2,6-diphenyl derivatives, in order to fully quantify the relative electronic effects resulting from benzannulation of the parent DTP building block. Such comparative analysis reveals that benzannulation results in a red-shifted absorbance, but to a lesser extent than simple phenyl-capping of the DTP. More surprising is that benzannulation results in stabilization of the BBTP HOMO, compared to the destabilization normally observed with extending the conjugation length of the backbone.

## 1. Introduction

Conjugated organic systems can provide materials that exhibit the electronic and optical properties of inorganic semiconductors, while retaining various attractive properties typically associated with organic plastics, including mechanical flexibility and low costs for production [1,2,3]. While such organic semiconductors are typically viewed as very modern materials, the study of these polymers dates as far back as the early 19th century [4,5,6], although significant interest did not arise until the first reports of their conductive nature in the early 1960s [6,7,8,9,10]. Since that point, the continuing academic and technological interest in these materials has given rise to the current field of organic electronics. Such development of technological applications has focused on organic photovoltaics (OPVs), field effect transistors (FETs), organic light-emitting diodes (OLEDs), electrochromic devices, and sensors [1,2,3].

**Figure 1:**
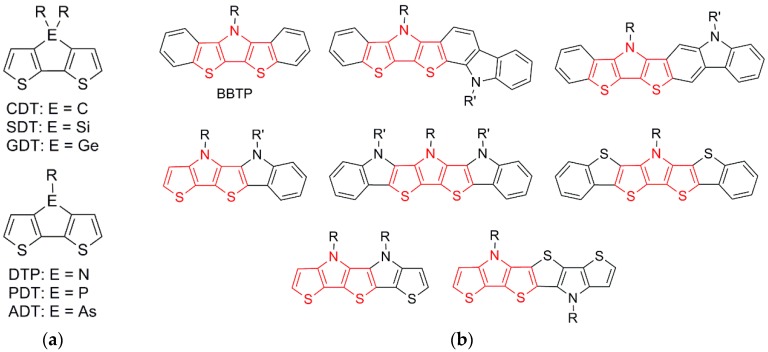
(**a**) Fused 2,2′-bithiophene building blocks; (**b**) Various extended fused-ring analogues of dithieno[3,2-*b*:2′,3′-*d*]pyrrole (DTP).

One of the powerful aspects of these materials has been the ability to tune their electronic and optical properties at the molecular level via synthetic modification. This is especially true for thiophene-based materials, in which the ease and versatility of form via synthetic manipulation has made them especially popular [2,3]. A particularly popular approach for the synthetic modification of thiophene-based materials has been the application of fused-ring building blocks, especially the various fused 2,2′-bithiophenes shown in Figure 1a, including cyclopenta[2,1-*b*:3,4-*b*’]dithiophene (CDT) [2,3,11,12,13], silolo[3,2-*b*:4,5-*b*’]dithiophene (SDT) [3,11,12], dithieno[3,2-*b*:2′,3′-*d*]pyrrole (DTP) [2,3,11,12,13,14,15,16], phospholo[3,2-*b*:4,5-*b*’]dithiophene (PDT) [3,11,12], germolo[3,2-*b*:4,5-*b*’]dithiophene (GTP) [3,17,18,19], and arsolo[3,2-*b*:4,5-*b*’]dithiophene (ADT) [20,21,22]. The fused-ring nature of these compounds provides a rigid, planar ground state, which can lead to improved conjugation, increased delocalization, and decreased energy gaps, while also reducing interannular torsional vibrations, thus contributing to increased emission quantum yields. In addition, the bridging unit (ER or ER_2_ in Figure 1a) can contribute via inductive effects to tune the corresponding HOMO or LUMO energies, while the central placement of the side chains allows the use of fairly bulky groups without the introduction of unwanted steric interactions that can lead to reduced backbone planarity in the resulting materials [12,13].

More recently, various groups have begun investigating analogous building blocks incorporating further annulated rings. This is especially true for the popular DTP units, for which a number of such extended analogues have been reported (Figure 1b) [23,24,25,26,27,28,29,30,31,32]. Of these extended analogues, the oldest and most simple are the bis[1]benzothieno[3,2-*b*:2′,3′-*d*]pyrroles (BBTPs), which were first introduced by Liu in 2008 [23]. In the following decade, additional reports of BBTPs have appeared [24,25,26,27], but the scope of these building blocks is still somewhat limited, with only a total of seven members reported to date. It should be pointed out that the nomenclature used to refer to these units changes from paper to paper, none of which correspond to the correct and proper IUPAC or CAS nomenclature. This is perhaps not surprising as the nomenclature of such complicated species is rarely covered in even graduate coursework, such that most in the field are not very familiar with the subtleties of correctly naming fused-ring systems. In order to assist the field with such complexities, an educational guide to fused-ring nomenclature has been recently published [33].

Although BBTPs have been studied as both molecular materials [23,24] and incorporated into polymeric materials [26,34], only four BBTPs have currently been characterized in terms of their optical and electronic properties [23,24]. Perhaps more critically, at no point have the resulting properties of BBTPs been directly compared to the parent DTPs, although some limited attempts to compare analogous polymeric materials have been reported [23]. In order to provide a more detailed characterization of these fused-ring units, as well as increase the scope of BBTPs, the synthesis and characterization of four N-substituted BBTPs are reported herein. Of these four BBTPs, three are previously unreported, and one is only the second example of an *N*-arylBBTP. Lastly, the optical, electronic, and structural properties of the BBTP units have been compared to the parent *N*-alkyl- and *N*-aryl-DTPs, as well as their corresponding 2,6-diphenyl derivatives, in order to fully quantify the relative electronic effects resulting from benzannulation of the parent DTP building block.

## 2. Results and Discussion

### 2.1. Synthesis

**Scheme 1:**
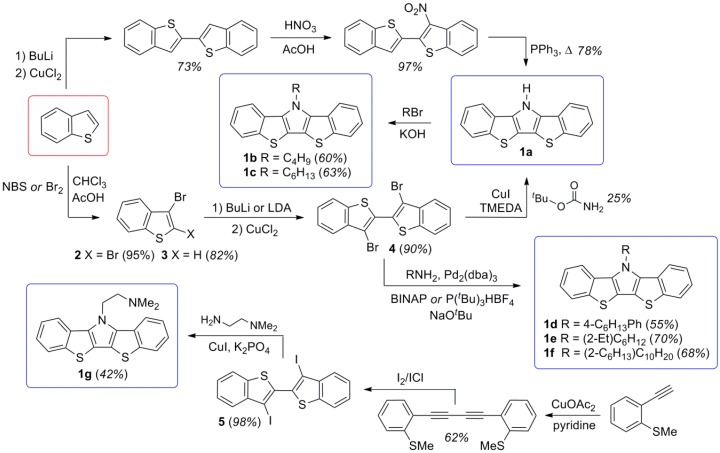
Previously reported synthetic routes to bis[1]benzothieno[3,2-*b*:2′,3′-*d*]pyrroles (BBTPs).

To date, several different approaches have been utilized for the synthesis of BBTPs, as outlined in Scheme 1. Although the earliest methods involved the cyclization of 3-nitro-2,2′-dibenzo[*b*]thiophene to give the unfunctionalized BBTP **1a [23]**, which could then be *N*-functionalized, the majority of the known synthetic methods utilize a 3,3′-dihalo-2,2′-bi(benzo[*b*]thiophene) (either **4** or **5**) from which the central pyrrole ring is formed via catalytic amination [24,25,26,27]. The intermediate **4** is not only the most common route to BBTPs, but also to other benzannulated species [35,36,37,38,39,40] such as thieno[3,2-*b*:4,5-*b*’]bis[1]benzothiophene [36,38,40] and phospholo[3,2-*b*:4,5-*b*’]bis[1]benzothiophene [37].

**Scheme 2:**
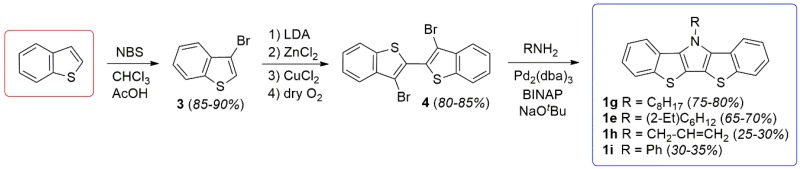
Optimized general synthesis of bis[1]benzothieno[3,2-*b*:2′,3′-*d*]pyrroles (BBTPs).

In the synthetic efforts reported here, BBTPs were produced via analogy with the more well-developed DTPs (Scheme 2) [13]. This begins with the synthesis of 3-bromobenzo[*b*]thiophene (**3**), which can be reproducibly generated in high yield via previously reported methods [23]. Precursor **3** can then be dimerized to generate the critical intermediate **4** via methods our group had previously optimized for generation of the analogous 3,3′-bromo-2,2′-bithiophene [15]. These methods allow the production of **4** as a high purity, white crystalline solid. 

**Figure 2:**
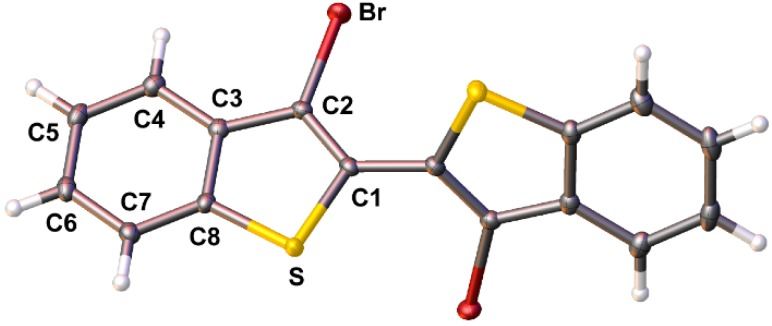
Ellipsoid plot of 3,3′-dibromo-2,2′-bi(benzo[*b*]thiophene) (**4**) at the 50% probability level.

It should be highlighted that previous reports have claimed the high yield (ca. 90%) production of **4** from either **3** or 2,3-dibromobenzo[*b*]thiophene (**2**) via more simple methods [24,25,26]. However, these reports either provide no synthetic procedure or details to support the claim [25,26], or the reported data is indicative of material of low purity, with one such report stating **4** to be a “purple brown solid” [24]. Most reports give more realistic yields of 40–56% via these basic methods [35,36,37,38,39]. In the optimized methods reported here, formation of the copper intermediate is facilitated by initial transmetallation with ZnCl_2_, followed by sequential reaction with CuCl_2_ [15,41]. Oxidative coupling of the copper intermediate is then assisted with the addition of dry O_2_, to allow the production of **4** in high yield (80–85%). In addition, the purity of **4** generated via these methods is high enough that X-ray quality crystals were easily obtained, thus allowing its structural determination (Figure 2). 

The intermediate **4** can then be smoothly transformed into the BBTP products via a double C-N bond formation through Buchwald-Hartwig amination. This was accomplished using standard conditions previously developed for DTPs, using a palladium pre-catalyst coupled with the chelating diphosphine ligand 2,2′-bis(diphenylphosphino)-1,1′-binaphthyl (BINAP) [13]. The use of conventional long chain alkylamines resulted in BBTPs in yields of 65–80%, while the application of aniline and allylamine gave reduced yields. In the case of aniline, it is believed that the amination proceeds smoothly, but the resulting BBTP **1i** is quite rigid and tends to aggregate, which complicates purification and leads to lower isolated yields. The final application of allylamine is complicated by the very low boiling point of the small molar mass amine (55–58 °C), thus causing loss of the volatile reagent during the high temperature amination step that leads to quite low yields. This could potentially be overcome through the future use of sealed pressurized conditions. 

### 2.2. X-ray Crystallography and Structural Analysis of BBTPs

**Figure 3:**
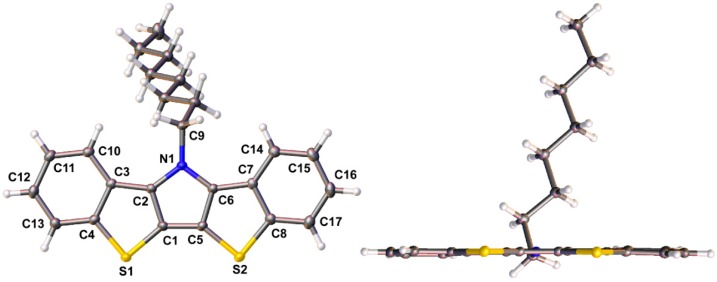
Face and edge ellipsoid plots of *N*-octylbis[1]benzothieno[3,2-*b*:2′,3′-*d*]pyrrole **1g** at the 50% probability level.

**Figure 4:**
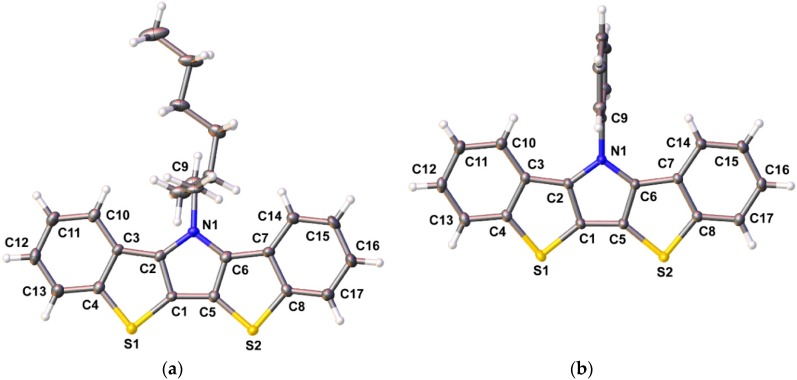
Ellipsoid plots of bis[1]benzothieno[3,2-*b*:2′,3′-*d*]pyrroles **1e** (**a**) and **1i** (**b**) at the 50% probability level.

The rigid, extended fused-ring structure of the BBTP compounds make them highly crystalline, and the crystal structures have been determined for three of the four BBTPs reported here. Ellipsoid plots of BBTPs **1g**, **1e**, and **1i** are shown in Figure 3 and Figure 4. As can be seen for BBTP **1g** in Figure 3, the backbones of these structures are extremely flat with deviations of only ca. 1° from planarity.

Selected bond distances for these three BBTPs are given in Table 1, along with those of the previously reported BBTPs **1a**, **1c**, **1d**, and *N*-octylDTP for comparison. The fused-ring structures of all six BBTP compounds show excellent agreement, with deviations of ca. 0.01 Å or less for each bond. A slightly greater difference is observed in the bond between the pyrrole and the corresponding side chain (i.e., N1-C9), in which the two aryl derivatives exhibit a shortening of ca. 0.03 Å in comparison to the alkyl derivatives. This difference is less than that observed in the analogous DTP compounds, however, which can potentially be attributed to differences in the orientation of the phenyl rings relative to the fused ring backbone. While the *N*-phenyl ring of DTPs exhibits a dihedral angle of ca. 46° relative to the DTP backbone [14], the *N*-phenyl rings of the analogous BBTPs is nearly perpendicular (ca. 85°). This difference is most likely due to increased sterics between the *ortho*-hydrogens on the *N*-phenyl ring and the C-H bonds at the 1- and 10-positions of the BBTP backbone. The enhanced dihedral angle would further limit conjugation between the phenyl ring and the fused-ring backbone, thus leading to elongation of the N-C bond. As such, one would also expect less electronic differences between the *N*-alkyl- and *N*-aryl-BBTPs as a result.

In comparison to *N*-octylDTP as a representative example, the BBTP units exhibit elongation of nearly all of the bonds that make up their central DTP core. As previously reported [14], the parent DTPs incorporate a central pyrrole that agrees well with the isolated heterocycle and this central ring then seems to force the fused thiophenes to match its geometry. In contrast, the bond lengths of the BBTP pyrrole deviate from that of the isolated heterocycle, and the structure is now much more consistent with that of dibenzothiophene [42]. For example, the fused bonds C1-C2 (1.396 Å) and C3-C4 (1.420 Å), of **1g** more closely match the fused bond of dibenzothiophene (1.409 Å) [42] than either that of isolated thiophene (1.370 Å) or pyrrole (1.382 Å) [43]. As such, the greater aromaticity of the terminal benzene rings now seems to dictate the overall geometry of the BBTP backbone. The primary exception to this general bond length elongation is seen in the central bond between the thiophenes, C1-C5, which is slightly shorter than that observed in DTP (ca. 1.41 vs. 1.42 Å). This bond is also shorter than the interannular bonds of either 2,2′-bithiophene (1.45 Å) [44] or **4** (1.458 Å), implying strong coupling through the central pyrrole ring.

### 2.3. Absorption Spectroscopy

**Figure 5:**
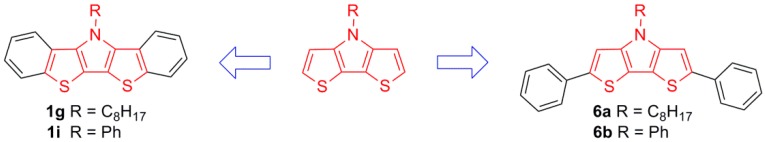
Analogous DTP-based species utilized to compare the relative effects of benzannulation verses simple phenyl substitution.

**Figure 6:**
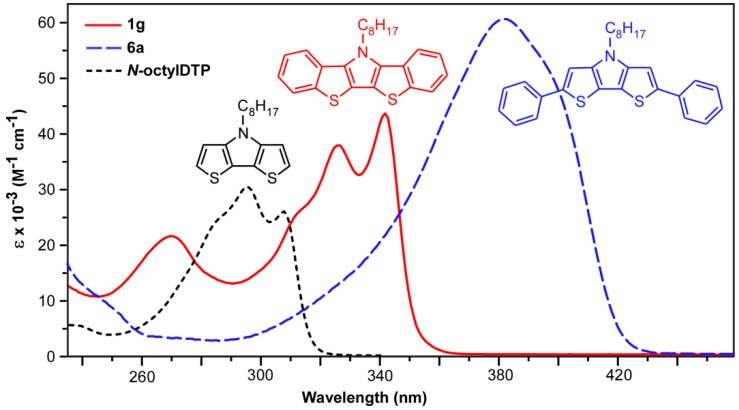
UV-visible spectra of *N*-octylbis[1]benzothieno[3,2-*b*:2′,3′-*d*]pyrrole (**1g**), *N*-octyl-2,6-diphenyldithieno[3,2-*b*:2′,3′-*d*]pyrrole (**6a**), and *N*-octyldithieno[3,2-*b*:2′,3′-*d*]pyrrole in CH_3_CN.

One of the goals of the current study was to fully quantify the relative electronic effects resulting from benzannulation of the parent DTP building block. As such, the photophysical data of the BBTPs were directly compared to both the parent *N*-octyl- and *N*-phenyl-DTPs, and to their 2,6-diphenyl derivatives (Figure 5). Photophysical data for all species are collected in Table 2 and representative UV-vis spectra of **1g**, **6a**, and *N*-octylDTP are shown in Figure 6.

**Figure 7:**
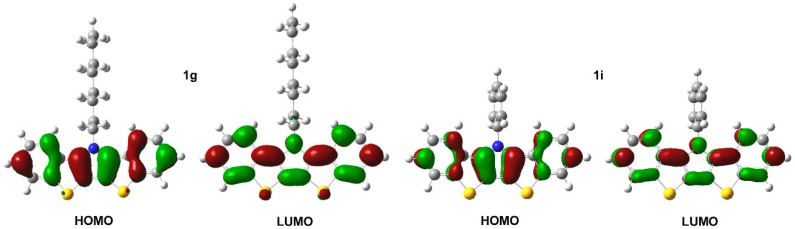
Electronic density contours, calculated at B3LYP/6-31G(d,p) level, for the HOMO and LUMO frontier molecular orbitals of bis[1]benzothieno[3,2-*b*:2′,3′-*d*]pyrroles **1g** and **1i**.

The BBTP series all exhibit two closely-spaced transitions at ca. 341 and 325 nm, as well as a higher energy shoulder at ca. 311 nm. Due to the close energetic spacing of these transitions, it is logical to assign these as vibrational components of a single electronic transition. This is further supported by the fact that the observed spacings of ca. 1400 cm^−1^ are in close agreement with the breathing modes of functionalized thiophenes (1354–1422 cm^−1^) and pyrroles (1415–1491 cm^−1^) [45]. A separate electronic transition is also observed at higher energy (ca. 266 nm). The extinction coefficients for all of these transitions fall roughly between 20,000–50,000 M^−1^ cm^−1^, with the coefficients of the low energy transition nearly twice that of the parent DTPs, consistent with the increase in the cross-sectional area of the BBTP relative to DTP. All of these transitions are assigned as simple π→π* transitions, which agree with the calculated electron density distributions of the HOMO and LUMO shown in Figure 7, and the measured extinction coefficients correspond to strongly allowed transitions. The energies of these transitions are essentially independent of the nature of the N-substitution, similar to that of DTPs [14]. This is consistent with the frontier orbitals shown in Figure 7, which show that the N-substituents reside at a node in the BBTP HOMO, and the side chain contributes to neither the HOMO or LUMO, both of which are similar to previous calculations of DTP [13,14,15].

In addition to the enhanced absorbance intensities, the BBTP spectra are also red-shifted by ca. 30 nm in comparison to the parent DTPs, consistent with the greater conjugation length resulting from benzannulation of the DTP backbone. As shown in Figure 6, however, the effect of this benzannulation is limited in comparison to that resulting from end-capping the DTP with phenyl rings. The corresponding red-shift resulting from the phenyl end-capping is more than twice that of benzannulation, with a corresponding increase in absorbance intensity. Although the phenyl-capped DTPs **6a** and **6b** consists of an additional four π-electrons and a larger cross-sectional area, this is not enough to account for the drastic difference in absorption energies. Nevertheless, this suggests that benzannulation of such fused-ring heterocycles is not as effective as simple aryl-functionalization in extending the delocalization of the conjugated backbone.

### 2.4. Electrochemistry

**Figure 8:**
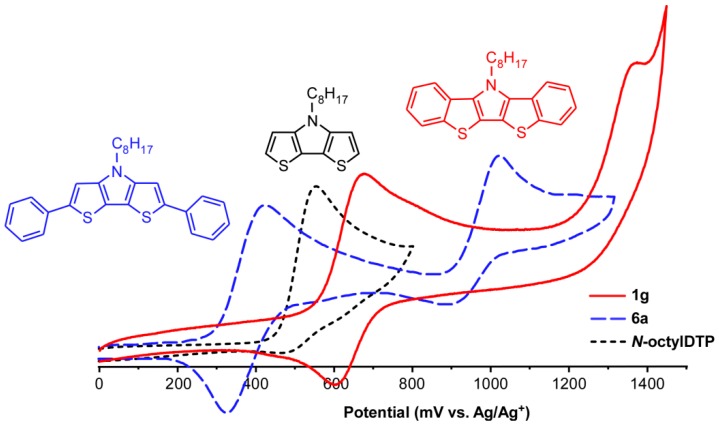
Cyclic voltammograms of *N*-octylbis[1]benzothieno[3,2-*b*:2′,3′-*d*]pyrrole (**1g**), *N*-octyl-2,6-diphenyldithieno[3,2-*b*:2′,3′-*d*]pyrrole (**6a**), and *N*-octyldithieno[3,2-*b*:2′,3′-*d*]pyrrole.

In order to determine the effects of DTP benzannulation on the frontier orbitals of the resulting BBTPs, the electrochemistry of the BBTP series was investigated by cyclic voltammetry (CV) and compared to the respective DTP parent species, as well as the analogous diphenyl-capped DTPs **6a** and **6b**. Representative voltammograms of **1g**, **6a**, and *N*-octylDTP are shown in Figure 8, and the collected CV data is given in Table 3. All BBTPs exhibit an initial well-defined, quasi-reversible redox couple, followed by a second irreversible oxidation. Similar to previously reported DTPs [13,14,15], the potential of the initial oxidation is dependent on the nature of the *N*-functionalization, with *N*-arylBBTPs exhibiting oxidation at slightly higher potentials. However, this effect is significantly diminished in the BBTPs, which exhibit shifts of only 50 mV in comparison to shifts of ca. 90–100 mV for the analogous DTPs. This difference is attributed to the nearly orthogonal orientation of the phenyl rings observed in the X-ray structures of *N*-arylBBTPs, which would severely limit conjugation between the phenyl ring and the fused-ring backbone. As such, the electronic effect of the *N*-aryl functionalization is assumed to be predominately inductive.

Although the benzannulation of DTP extends the conjugated backbone, the first oxidation of the BBTPs actually occurs at higher potentials than the analogous DTPs. In fact, the initial oxidation of the *N*-alkylBBTPs is essentially the same as the more stabilized *N*-arylDTPs. In comparison, the addition of phenyl end-caps to DTP results in the expected lowering of the potential for the first oxidation. Thus, the modulation of the frontier orbitals by benzannulation is directly opposite that of simple addition of phenyl groups. This seemingly contradictory electronic stabilization with extension of the π-backbone must be at least partially related to the structural changes observed in the DTP core upon benzannulation.

## 3. Materials and Methods

*N*-Octyldithieno[3,2-*b*:2′,3′-*d*]pyrrole [47], *N*-phenyldithieno[3,2-*b*:2′,3′-*d*]pyrrole [16], *N*-octyl-2,6-diphenyldithieno[3,2-*b*:2′,3′-*d*]pyrrole [16], *N*-phenyl-2,6-diphenyldithieno[3,2-*b*:2′,3′-*d*]pyrrole [16], and 3-bromobenzo[*b*]thiophene (**3**) [23] were prepared as previously reported. Dry THF and xylenes were obtained via distillation over sodium/benzophenone. Dry CH_3_CN was obtained via distillation over CaH_2_. All other chemical species were reagent grade and used without further purification. All reactions were carried out under an inert atmosphere. All glassware for chemical synthesis was oven-dried, assembled while still hot, and cooled under dry nitrogen. ^1^H and ^13^C NMR spectra were collected on a 400 MHz spectrometer (400 MHz ^1^H, 100 MHz ^13^C) in CDCl_3_ and referenced to the CHCl_3_ signal. Peak multiplicity is reported as follows: s = singlet, d = doublet, t = triplet, quint = quintet, sept = septet, dd = doublet of doublets, dt = doublet of triplets, ddt = doublet of doublet of triplets, m = multiplet. See ESI for copies of all NMR spectra.

### 3.1. Synthesis of 3,3′-Dibromo-2,2′-bi(benzo[b]thiophene) (**4**)

The following is a modification of previously reported methods for the synthesis of 3,3′-dibromo-2,2′-bithiophene [15]. To a 250 mL 3-necked flask was added THF (120 mL), which was then cooled to 0 °C. Diisopropylamine (3.9 mL, 27.5 mmol) was added, followed by BuLi (11.0 mL, 2.5 M in hexanes, 27.5 mmol), and the mixture allowed to stir for 30 min. 3-Bromobenzo[*b*]thiophene (5.32 g, 25.0 mmol) was added and the solution was stirred for another 2 h. ZnCl_2_ (3.75 g, 27.5 mmol) was added in one portion and stirred for 15 min. The solution was cooled to −78 °C and CuCl_2_ (3.70 g, 25.0 mmol) was added in one portion, and stirred for 30 min. Dry O_2_ was bubbled through the solution for 2 min, and the reaction was stirred until completion (as monitored by TLC, ~1 h). The reaction was then warmed to room temperature and quenched with saturated aqueous NH_4_Cl. The organic layer was separated, and the aqueous layer was extracted with diethyl ether. The combined organic layers were dried with MgSO_4_, concentrated via rotary evaporation, and purified by silica gel chromatography (hexanes) to give the isolated product as a white solid (80–85%). mp 169.0–171.0 °C (lit. mp 178 °C [35]); ^1^H NMR (CDCl_3_) δ 7.93 (dd, *J* = 1.2, 7.2 Hz, 2H), 7.87 (dd, *J* = 1.2, 7.2 Hz, 2H), 7.53 (dt, *J* = 1.2, 7.2 Hz, 2H), 7.49 (dt, *J* = 1.2, 7.2 Hz, 2H); ^13^C NMR (CDCl_3_) δ 139.2, 138.1, 129.4, 126.4, 125.5, 124.1, 122.3, 110.9. NMR data agree well with previously reported values [35,36,37,38,39,40].

### 3.2. General Synthesis of N-Functionalized Bis[1]benzothieno[3,2-b:2′,3′-d]pyrroles

To a 50 mL 3-necked flask was added compound **4** (0.424 g, 1.0 mmol), sodium *tert*-butoxide (0.233 g, 2.4 mmol), Pd_2_(dba)_3_ (0.046 g, 0.050 mmol), and BINAP (0.062 g, 0.10 mmol), followed by evacuation of the flask and backfilling with nitrogen. Xylenes (15 mL) was then added, followed by the chosen amine (1.1 mmol), and the reaction mixture was heated to slightly under reflux (ca. 138 °C) with stirring for overnight. The reaction was then cooled to room temperature, water was added, and the mixtureextracted with chloroform. The combined organic layers were dried with MgSO_4_, filtered, concentrated via rotary evaporation, and purified by silica gel chromatography, to give the isolated product as a crystalline solid.

#### 3.2.1. *N*-Octylbis[1]benzothieno[3,2-*b*:2′,3′-*d*]pyrrole (**1g**)

The crude material was eluted with hexanes to give **1g** as a white solid (75–80% yield). mp 93.5–94.0 °C; ^1^H NMR (CDCl_3_) δ 7.91 (d, *J* = 8.0 Hz, 2H), 7.89 (d, *J* = 8.0 Hz, 2H), 7.42 (t, *J* = 8.0 Hz, 2H), 7.29 (t, *J* = 8.0 Hz, 2H), 4.80 (t, *J* = 7.6 Hz, 2H), 2.06 (quint, *J* = 7.6 Hz, 2H), 1.53 (quint, *J* = 7.6 Hz, 2H), 1.37 (quint, *J* = 7.6 Hz, 2H), 1.28–1.21 (m, 6H), 0.86 (t, *J* = 7.6 Hz, 3H); ^13^C NMR (CDCl_3_) δ 141.9, 137.5, 127.5, 124.5, 124.4, 123.1, 118.8, 114.5, 47.7, 32.0, 31.3, 29.6, 29.3, 27.0, 22.8, 14.3.

#### 3.2.2. *N*-(2-Ethylhexyl)bis[1]benzothieno[3,2-*b*:2′,3′-*d*]pyrrole (**1e**)

The crude material was eluted with hexanes to give **1e** as a white solid (65–70% yield). mp 148.2–149.0 °C (lit. mp 147–148 °C [25]); ^1^H NMR (CDCl_3_) δ 7.92 (d, *J* = 8.0 Hz, 2H), 7.86 (d, *J* = 8.0 Hz, 2H), 7.42 (t, *J* = 8.0 Hz, 2H), 7.30 (t, *J* = 8.0 Hz, 2H), 4.70 (dd, *J* = 8.0, 14.0 Hz, 1H), 4.66 (dd, *J* = 8.0, 14.0 Hz, 1H), 2.24 (sept, *J* = 7.6 Hz, 2H), 1.60–1.20 (m, 8H), 0.90 (t, *J* = 7.6 Hz, 3H), 0.84 (t, *J* = 7.6 Hz, 3H); ^13^C NMR (CDCl_3_) δ 141.9, 137.7, 127.6, 124.5, 124.2, 123.1, 119.0, 114.6, 52.1, 39.2, 31.8, 31.7, 25.0, 22.8, 14.3, 11.1. NMR data agree well with previously reported values [25].

#### 3.2.3. *N*-Allylbis[1]benzothieno[3,2-*b*:2′,3′-*d*]pyrrole (**1h**)

The crude material was eluted with 1% ethyl acetate in hexanes to give **1h** as a white solid (25–30% yield). ^1^H NMR (CDCl_3_) δ 7.88 (dd, *J* = 0.92, 7.4 Hz, 2H), 7.86 (dd, *J* = 0.92, 7.4 Hz, 2H), 7.42 (dt, *J* = 0.92, 7.4 Hz, 2H), 7.32 (dt, *J* = 0.92, 7.4 Hz, 2H), 6.28 (ddt, *J* = 4.4, 10.7, 16.9 Hz, 1H), 5.44 (dt, *J* = 1.8, 4.4 Hz, 2H), 5.29 (ddt, *J* = 0.9, 1.8, 10.7 Hz, 1H), 5.18 (ddt, *J* = 0.9, 1.8, 16.9 Hz, 1H); ^13^C NMR (CDCl_3_) δ 142.0, 137.9, 132.8, 127.5, 124.6(1), 124.5(8), 123.5, 119.1, 117.6, 115.1, 49.4.

#### 3.2.4. *N*-Phenylbis[1]benzothieno[3,2-*b*:2′,3′-*d*]pyrrole (**1i**)

The crude material was eluted with hexanes to give **1i** as a white solid (30–35% yield). mp 201.6–202.8 °C; ^1^H NMR (CDCl_3_) δ 7.83 (d, *J* = 8.0 Hz, 2H), 7.72–7.65 (m, 5H), 7.23 (d, *J* = 8.0 Hz, 2H), 7.16 (t, *J* = 8.0 Hz, 2H), 7.12 (t, *J* = 8.0 Hz, 2H); ^13^C NMR not obtained due to aggregation at suitable concentrations.

### 3.3. X-ray Crystallography

X-ray quality crystals of **4**, **1g**, **1e**, and **1i** were grown by the slow evaporation of a hexane solution. The X-ray intensity data of the crystals were measured at 100 K on a Bruker Kappa Apex II Duo CCD-based X-ray diffractometer system equipped with a Mo-target X-ray tube (λ = 0.71073 Å) operated at 2000 W of power. The detector was placed at a distance of 5.000 cm from the crystal and data collected via the Bruker APEX2 software package. The frames were integrated with the Bruker SAINT software package. The unit cell was determined and refined by least-squares upon the refinement of XYZ-centeroids of reflections above 20σf(I). The structure was refined using the Bruker SHELXTL (Version 5.1) Software Package.

Crystal data for **4** (C_16_H_8_Br_2_S_2_, M = 424.16 g/mol): triclinic, space group P-1, a = 7.4904(2) Å, b = 7.6660(2) Å, c = 14.4986(3) Å, α = 102.0470(10)°, β = 91.9550(10)°, γ = 118.3950(10)°, *V* = 707.58(3) Å^3^, *Z* = 2, T = 100(2) K, μ = 6.007 mm^−1^, D_calc_ = 1.991 g/cm^3^, 25256 reflections measured, 3377 unique (R_int_ = 0.0157) which were used in all calculations. The final R_1_ was 0.0138 (I > 2σ(I)) and wR_2_ was 0.0357 (all data).

Crystal data for **1g** (C_24_H_25_NS_2_, M = 391.57 g/mol): monoclinic, space group P2(1)/n, a = 5.5843(4) Å, b = 16.9653(12) Å, c = 20.5440(15) Å, β = 92.396(1)°, *V* = 1944.6(2) Å^3^, *Z* = 4, T =100(2) K, μ = 0.283 mm^−1^, D_calc_ = 1.337 g/cm^3^, 19766 reflections measured, 4644 unique (R_int_ = 0.0307) which were used in all calculations. The final R_1_ was 0.0325 (I > 2σ(I)) and wR_2_ was 0.0832 (all data).

Crystal data for **1e** (C_24_H_25_NS_2_, M = 391.57 g/mol): triclinic, space group P-1, a = 8.6365(6) Å, b = 10.7755(8) Å, c = 11.1074(8) Å, α = 94.297(3)°, β = 105.748(3)°, γ = 92.076(3)°, *V* = 990.36(12) Å^3^, *Z* = 2, T = 100(2) K, μ = 0.278 mm^−1^, D_calc_ = 1.313 g/cm^3^, 28835 reflections measured, 3509 unique (R_int_ = 0.0533) which were used in all calculations. The final R_1_ was 0.0784 (I > 2σ(I)) and wR_2_ was 0.2361 (all data).

Crystal data for **1i** (C_22_H_13_NS_2_, M = 355.45 g/mol): monoclinic, space group P2(1)/n, a = 16.2108(5) Å, b = 10.6136(3) Å, c = 20.8222(6) Å, β = 110.374(2)°, *V* = 3358.44(17) Å^3^, *Z* = 8, T =100(2) K, μ = 0.320 mm^−1^, D_calc_ = 1.406 g/cm^3^, 68878 reflections measured, 10272 unique (R_int_ = 0.0224) which were used in all calculations. The final R_1_ was 0.0339 (I > 2σ(I)) and wR_2_ was 0.0928 (all data).

CCDC 1861451–1861454 contains the supplementary crystallographic data for this paper. These data can be obtained free of charge via http://www.ccdc.cam.ac.uk/conts/retrieving.html (or from the CCDC, 12 Union Road, Cambridge CB2 1EZ, UK; Fax: +44 1223 336033; E-mail: deposit@ccdc.cam.ac.uk).

### 3.4. Absorption Spectroscopy

Spectra were measured on a dual beam scanning UV-visible-NIR spectrophotometer in 1 cm quartz cuvettes. Samples were prepared as dilute CH_3_CN solutions. Extinction coefficients were determined via standard Beer-Lambert relationships using at least five standard solutions of different concentrations.

### 3.5. Computational Modeling

Calculations were performed with the Gaussian 09 program [48]. The molecular geometries of the neutral states were calculated at the DFT level using the B3LYP functional [49,50] and the 6-31G(d,p) basis set. A single point energy calculation was then performed using methods matching the geometry optimization, from which molecular orbital diagrams were generated.

### 3.6. Electrochemical Measurements

All electrochemical methods were performed utilizing a three-electrode cell consisting of a platinum disc working electrode, a platinum wire auxiliary electrode, and a Ag/Ag^+^ reference electrode (0.251 V vs. SCE) [51]. Supporting electrolyte consisted of 0.10 M TBAPF_6_ in dry CH_3_CN. Solutions were deoxygenated by sparging with argon prior to each scan and blanketed with argon during the measurements. All measurements were collected at a scan rate of 100 mV/s. E_HOMO_ values were estimated from the oxidation in relation to ferrocene (60 mV vs. Ag/Ag^+^), using the value of 5.1 eV vs. vacuum for ferrocene [46].

## 4. Conclusions

An optimized synthesis of N-functionalized BBTPs has been developed and applied to the synthesis of four benzannulated building blocks. The optical, electronic, and structural properties of the resulting BBTP units have been compared to the parent *N*-alkyl- and *N*-aryl-DTPs, as well as their corresponding 2,6-diphenyl derivatives, in order to fully quantify the relative electronic effects resulting from benzannulation of the DTP core. Although benzannulation does increase the conjugation length of the fused ring species, resulting in a corresponding red shift in absorption, this is not as effective as simple end-capping with aryl groups. In terms of modulation of the frontier orbitals, however, benzannulation results in stabilization of the BBTP HOMO, compared to the destabilization normally observed with extending the conjugation length of the backbone. Thus, while benzannulation is less effective in extending delocalization and shifting absorption to lower energy, it is an effective approach to stabilizing the HOMO of strongly electron-rich units.

## Figures and Tables

**Table 1:** Selected bond lengths (Å) for various bis[1]benzothieno[3,2-*b*:2′,3′-*d*]pyrroles and *N*-octyldithieno[3,2-*b*:2′,3′-*d*]pyrrole.

Bond	1a ^1^	1c ^1^	1g	1e	1i	1d ^2^	*N*-octylDTP ^3^
S1-C1	1.732(2)	1.7362(16)	1.7338(14)	1.731(6)	1.7319(11)	1.7325(13)	1.719(3)
S1-C4	1.759(3)	1.7582(17)	1.7560(14)	1.763(6)	1.7546(12)	1.7536(14)	1.716(3)
C1-C2	1.389(3)	1.3974(19)	1.3960(18)	1.404(8)	1.3924(15)	1.3894(17)	1.384(4)
C2-N1	1.373(3)	1.384(2)	1.3842(17)	1.378(8)	1.3844(13)	1.3802(16)	1.379(5)
N1-C9		1.4706(16)	1.4650(16)	1.464(7)	1.4347(14)	1.4367(15)	1.451(4)
C2-C3	1.431(3)	1.431(2)	1.4382(19)	1.443(8)	1.4334(14)	1.4307(18)	1.416(5)
C3-C4	1.407(3)	1.422(2)	1.4201(19)	1.412(8)	1.4171(15)	1.4159(18)	1.349(6)
C1-C5	1.412(3)	1.411(2)	1.4080(19)	1.410(8)	1.4123(14)	1.4163(18)	1.420(4)
C3-C10	1.402(3)	1.404(2)	1.4079(18)	1.406(8)	1.4050(16)	1.3984(19)	
C4-C13	1.392(3)	1.394(2)	1.393(2)	1.387(9)	1.3991(15)	1.3951(19)	
C10-C11	1.372(4)	1.382(3)	1.384(2)	1.387(9)	1.3877(16)	1.386(2)	
C11-C12	1.396(4)	1.397(2)	1.399(2)	1.386(9)	1.4011(18)	1.389(2)	
C12-C13	1.384(4)	1.378(2)	1.386(2)	1.381(9)	1.3893(17)	1.384(2)	

^1^ Data from reference [23]. ^2^ Data from reference [24]. ^3^ Adapted from reference [14].

**Table 2:** UV-visible data for various bis[1]benzothieno[3,2-*b*:2′,3′-*d*]pyrroles and dithieno[3,2-*b*:2′,3′-*d*]pyrrole. ^1^

Compound	λ_max_ (nm)	ε (M^−1^ cm^−1^)	E_opt_ (eV) ^2^
*N*-octylDTP	309	26,100	3.88
	296	30,400	
*N*-phenylDTP	310	32,000	3.87
	299	35,200	
**1g**	341	44,300	3.52
	325	39,200	
	267	21,000	
**1e**	341	44,100	3.52
	325	39,100	
	267	20,900	
**1h**	340	48,000	3.52
	324	45,100	
	266	24,900	
**1i**	341	40,400	3.52
	325	36,700	
	265	22,500	
**6a**	381	61,000	2.95
**6b**	378	58,400	2.96

^1^ In CH_3_CN. ^2^ Calculated from the onset of absorption.

**Table 3:** Electrochemical data for various bis[1]benzothieno[3,2-*b*:2′,3′-*d*]pyrroles and dithieno[3,2-*b*:2′,3′-*d*]pyrroles. ^1^

Compound	E_1/2_^0/+1^ (V)	E (mV)	E_pa_^+1/+2^ (V)	E_HOMO_ (eV) ^2^
*N*-octylDTP	0.56 ^3^			−5.60
*N*-phenylDTP	0.65 ^3^			−5.69
**1g**	0.64	75	1.37	−5.68
**1e**	0.65	80	1.37	−5.69
**1h**	0.65	75	1.37	−5.69
**1i**	0.70	90	1.37	−5.70
**6a**	0.38	80	1.05	−5.42
**6b**	0.47	80	1.09	−5.51

^1^ All measurements in dry CH_3_CN containing 0.10 M TBAPF_6_. All potentials vs. Ag/Ag^+^. ^2^ E_HOMO_ = −(E_[ox vs. Fc+/Fc]_ + 5.1)(eV) [46]. ^3^ Irreversible, E_pa_ reported.

## Supplementary Materials

The electronic supplementary information (ESI) consisting of ^1^H and ^13^C NMR spectra for all compounds are available online.

## References

[1] Skotheim T.A., Reynolds J.R. (2007). Handbook of Conducting Polymers.

[2] Rasmussen S.C., Ogawa K., Rothstein S.D., Nalwa H.S. (2008). Synthetic Approaches to Band Gap Control in Conjugated Polymeric Materials. Handbook of Organic Electronics and Photonics.

[3] Perepichka I.F., Perepichka D.F. (2009). Handbook of Thiophene-Based Materials: Applications in Organic Electronics and Photonics.

[4] Rasmussen S.C. (2017). The Early History of Polyaniline: Discovery and Origins. Substantia.

[5] Rasmussen S.C. (2018). Revisiting the Early History of Synthetic Polymers: Critiques and New Insights. Ambix.

[6] Rasmussen S.C., Reynolds J.R., Skotheim T., Thompson B. (2018). Early history of Conjugated Polymers: From their Origins to the Handbook of Conducting Polymers. Handbook of Conducting Polymers.

[7] Rasmussen S.C., Strom E.T., Rasmussen S.C. (2011). Electrically Conducting Plastics: Revising the History of Conjugated Organic Polymers. 100+ Years of Plastics. Leo Baekeland and Beyond.

[8] Rasmussen S.C. (2014). The Path to Conductive Polyacetylene. Bull. Hist. Chem..

[9] Rasmussen S.C. (2015). Early History of Polypyrrole: The First Conducting Organic Polymer. Bull. Hist. Chem..

[10] Rasmussen S.C., Zhang Z., Rouabhia M., Moulton S.E. (2017). Early History of Conductive Organic Polymers. Conductive Polymers: Electrical Interactions in Cell Biology and Medicine.

[11] Baumgartner T. (2005). π-Conjugated Heterocyclic fused Bithiophene Materials. J. Inorg. Organomet. Polym. Mater..

[12] Rasmussen S.C., Evenson S.J., McCausland C.B. (2015). Fluorescent Thiophene-based Materials and Their Outlook for Emissive Applications. Chem. Commun..

[13] Rasmussen S.C., Evenson S.J. (2013). Dithieno[3,2-*b*:2′,3′-*d*]pyrrole-based Materials: Synthesis and Applications to Organic Electronics. Prog. Polym. Sci..

[14] Ogawa K., Rasmussen S.C. (2003). A Simple and Efficient Route to N-Functionalized Dithieno[3,2-*b*:2′,3′-*d*]- pyrroles: Fused-Ring Building Blocks for New Conjugated Polymeric Systems. J. Org. Chem..

[15] Evenson S.J., Seth C., Rasmussen S.C. (2010). *N*-Acyldithieno[3,2-*b*:2′,3′-*d*]pyrroles: Second Generation Dithieno[3,2-*b*:2′,3′-*d*]pyrrole Building Blocks with Stabilized Energy Levels. Org. Lett..

[16] Evenson S.J., Pappenfus T.M., Delgado M.C.R., Radke-Wohlers K., Navarrete J.T.L., Rasmussen S.C. (2012). Molecular Tuning in Highly Fluorescent Dithieno[3,2-*b*:2′,3′-*d*]pyrrole-based Oligomers: Effects of N-Functionalization and Terminal Aryl Unit. Phys. Chem. Chem. Phys..

[17] Amb C.M., Chen S., Graham K.R., Subbiah J., Small C.E., So F., Reynolds J.R. (2011). Dithienogermole As a Fused Electron Donor in Bulk Heterojunction Solar Cells. J. Am. Chem. Soc..

[18] Fei Z., Kim J.S., Smith J., Domingo E.B., Anthopoulos T.D., Stingelin N., Watkins S.E., Kim J.-S., Heeney M. (2011). A low band gap co-polymer of dithienogermole and 2,1,3-benzothiadiazole by Suzuki polycondensation and its application in transistor and photovoltaic cells. J. Mater. Chem..

[19] Gendron D., Morin P.-O., Berrouard P., Allard N., Aich B.R., Garon C.N., Tao Y., Leclerc M. (2011). Synthesis and Photovoltaic Properties of Poly(dithieno[3,2-*b*:2′,3′-*d*]germole) Derivatives. Macromolecules.

[20] Green J.P., Han Y., Kilmurray R., McLachlan M.A., Anthopoulos T.D., Heeney M. (2016). An Air-Stable Semiconducting Polymer Containing Dithieno[3,2-*b*:2′,3′-*d*]arsole. Angew. Chem. Int. Ed..

[21] Kato T., Imoto H., Tanaka S., Ishidoshiro M., Naka K. (2016). Facile synthesis and properties of dithieno-[3,2-*b*:2′,3′-*d*]arsoles. Dalton Trans..

[22] Green J.P., Cryer S.J., Marafie J., White A.J.P., Heeney M. (2017). Synthesis of a Luminescent Arsolo-[2,3-*d*:5,4-*d*′]bis(thiazole) Building Block and Comparison to Its Phosphole Analogue. Organometallics.

[23] Qi T., Guo Y., Liu Y., Xi H., Zhang H., Gao X., Liu Y., Lu K., Du C., Yu G., Zhu D. (2008). Synthesis and properties of the anti and syn isomers of dibenzothieno[*b,d*]pyrrole. Chem. Commun..

[24] Balaji G., Valiyaveettil S. (2009). Synthesis and Properties of Symmetric and Unsymmetric Dibenzothieno- pyrroles. Org. Lett..

[25] Balaji G., Della Pelle A.M., Popere B.C., Chandrasekaran A., Thayumanavan S. (2012). Synthesis and properties of thienopyrrole based heteroacenes – indolodibenzothienopyrrole and dicarbazolodithienopyrrole. Org. Biomol. Chem..

[26] Jung I.H., Kim J.-H., Nam S.Y., Lee C., Hwang D.-H., Yoon S.C. (2015). Development of New Photovoltaic Conjugated Polymers Based on Di(1-benzothieno)[3,2-*b*:2′,3′-*d*]pyrrole: Benzene Ring Extension Strategy for Improving Open-Circuit Voltage. Macromolecules.

[27] Gupta A., Flynn B.L. (2017). Linear and Angular Heteroacenes from Double-Electrophilic Cyclization (DEC) and DEC-Reductive Elimination of Diynes. Org. Lett..

[28] Gao P., Cho D., Yang X., Enkelmann V., Baumgarten M., Müllen K. (2010). Heteroheptacenes with Fused Thiophene and Pyrrole Rings. Chem. Eur. J..

[29] Balaji G., Phua D.I., Shim W.L., Valiyaveettil S. (2010). Synthesis and Characterization of Unsymmetric Indolodithienopyrrole and Extended Diindolodithienopyrrole. Org. Lett..

[30] Mitsudo K., Shimohara S., Mizoguchi J., Mandai H., Suga S. (2012). Synthesis of Nitrogen-Bridged Terthiophenes by Tandem Buchwald-Hartwig Coupling and Their Properties. Org. Lett..

[31] Wetzel C., Mishra A., Mena-Osteritz E., Liess A., Stolte M., Würthner F., Bäuerle P. (2014). Synthesis and Structural Analysis of Thiophene-Pyrrole-Based S,N-Heteroacenes. Org. Lett..

[32] Qin P., Kast H., Nazeeruddin M.K., Zakeeruddin S.M., Mishra A., Bäuerle P., Grätzel M. (2014). Low band gap S,N-heteroacene-based oligothiophenes as hole-transporting and light absorbing materials for efficient perovskite-based solar cells. Energy Environ. Sci..

[33] Rasmussen S.C. (2016). The Nomenclature of Fused-ring Arenes and Heterocycles: A Guide to an Increasingly Important Dialect of Organic Chemistry. Chem. Texts.

[34] Jung I.H., Kim J.-H., Nam S.Y., Lee C., Hwang D.-H., Yoon S.C. (2016). A di(1-benzothieno)-[3,2-*b*:2′,3′-*d*]pyrrole and isoindigo-based electron donating conjugated polymer for efficient organic photovoltaics. J. Mater. Chem. C.

[35] Dahlmann U., Neidlein R. (1997). The Diyne Reaction of 3,3′-Bis(phenylethynyl)-2,2′-bithiophene Derivatives via Rhodium Complexes: A Novel Approach to Condensed Benzo[2,l-*b*:3,4-*b*’]dithiophenes. Helv. Chim. Acta.

[36] Gao J., Li R., Li L., Meng Q., Jiang H., Li H., Hu W. (2007). High-Performance Field-Effect Transistor Based on Dibenzo[*d,d*′]thieno[3,2-*b*;4,5-*b*′]dithiophene, an Easily Synthesized Semiconductor with High Ionization Potential. Adv. Mater..

[37] Dienes Y., Eggenstein M., Kárpáti T., Sutherland T.C., Nyulászi L., Baumgartner T. (2008). Phosphorus-Based Heteropentacenes: Efficiently Tunable Materials for Organic n-Type Semiconductors. Chem.–Eur. J..

[38] Alessandrini L., Braga D., Jaafari A., Miozzo L., Mora S., Silvestri L., Tavazzi S., Yassar A. (2011). Optical Properties of Dibenzo[*d,d*′]thieno[3,2-*b*;4,5-*b*′]dithiophene Monocrystals: The Effect of Intermolecular Interactions. J. Phys. Chem. A.

[39] Zhao Y., Hao W., Ma W., Zang Z., Zhang H., Liu X., Zou S., Zhang H., Liu W., Gao J. (2014). Easily-soluble heteroacene bis(benzothieno)silole derivatives for sensing of nitro explosives. New J. Chem..

[40] Oechsle P., Paradies J. (2014). Ambidextrous Catalytic Access to Dithieno[3,2-*b*:2′,3′-*d*]thiophene (DTT) Derivatives by Both Palladium-Catalyzed C−S and Oxidative Dehydro C−H Coupling. Org. Lett..

[41] Kabir S.M.H., Miura M., Sasaki S., Harada G., Kuwatani Y., Yoshida M., Iyoda M. (2000). New Syntheses of Tricyclic Thiophenes and Cyclic Tetrathiophenes Using Transition-Metal-catalyzed Cyclization. Heterocycles.

[42] Katritzky A.R., Pozharskii A.F. (2000). Handbook of Heterocyclic Chemistry.

[43] Katritzky A.R., Pozharskii A.F. (2000). Handbook of Heterocyclic Chemistry.

[44] Barbarella G., Zambianchi M., Antolini L., Folli U., Goldoni F., Iarossi D., Schenetti L., Bongini A. (1995). Conformational properties of 3,3′-, 3,4′- and 4,4′-dimethyl- and -bis(methylsulfanyl)-2,2′-bithiophenes. J. Chem. Soc. Penkin Trans. 2.

[45] Katritzky A.R., Pozharskii A.F. (2000). Handbook of Heterocyclic Chemistry.

[46] Cardona C.M., Li W., Kaifer A.E., Stockdale D., Bazan G.C. (2011). Electrochemical Considerations for Determining Absolute Frontier Orbital Energy Levels of Conjugated Polymers for Solar Cell Applications. Adv. Mater..

[47] Evenson S.J., Mumm M.J., Pokhodnya K.I., Rasmussen S.C. (2011). Highly Fluorescent Dithieno[3,2-*b*:2′,3′-*d*]pyrrole-based Materials: Synthesis, Characterization and OLED Device Applications. Macromolecules.

[48] Frisch M.J., Trucks G.W., Schlegel H.B., Scuseria G.E., Robb M.A., Cheeseman J.R., Scalmani G., Barone V., Mennucci B., Petersson G.A. (2009). Gaussian 09, Revision A.02.

[49] Becke A.D. (1993). Density-functional thermochemistry. III. The role of exact exchange. J. Chem. Phys..

[50] Lee C.T., Yang W.T., Parr R.G. (1988). Development of the Colle-Salvetti correlation-energy formula into a functional of the electron density. Phys. Rev. B.

[51] Larson R.C., Iwamoto R.T., Adams R.N. (1961). Reference electrodes for voltammetry in acetonitrile. Anal. Chim. Acta.

