# Supplementary material for: Synthesis and Characterization of Bis[1]benzothieno[3,2-b:2′,3′-d]pyrroles: Quantitative Effects of Benzannulation on Dithieno[3,2-b:2′,3′-d]pyrroles
Source: Molecules. 2018 Sep 6;23(9):2279. doi: 10.3390/molecules23092279 (PMC6225175; doi:10.3390/molecules23092279)
Supplement: Supplementary file 1 [file molecules-23-02279-s001.pdf]

Synthesis and Characterization of Bis[1]benzothieno[3,2-*b*:2',3'-*d*]pyrroles: Quantitative Effects of Benzannulation on Dithieno[3,2-*b*:2',3'-*d*]pyrroles

Rylan M. W. Wolfe, Evan W. Culver, and Seth C. Rasmussen *

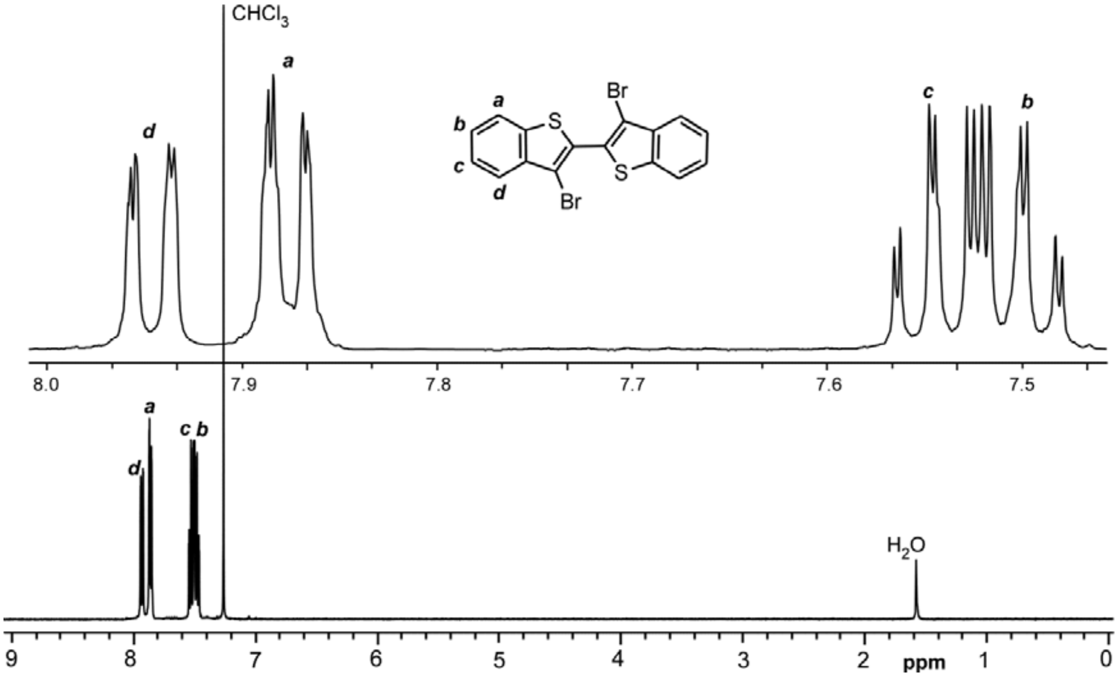

Figure S1. ^{1}H NMR Spectrum of 3,3′-dibromo-2,2′-bi(benzo[*b*]thiophene).

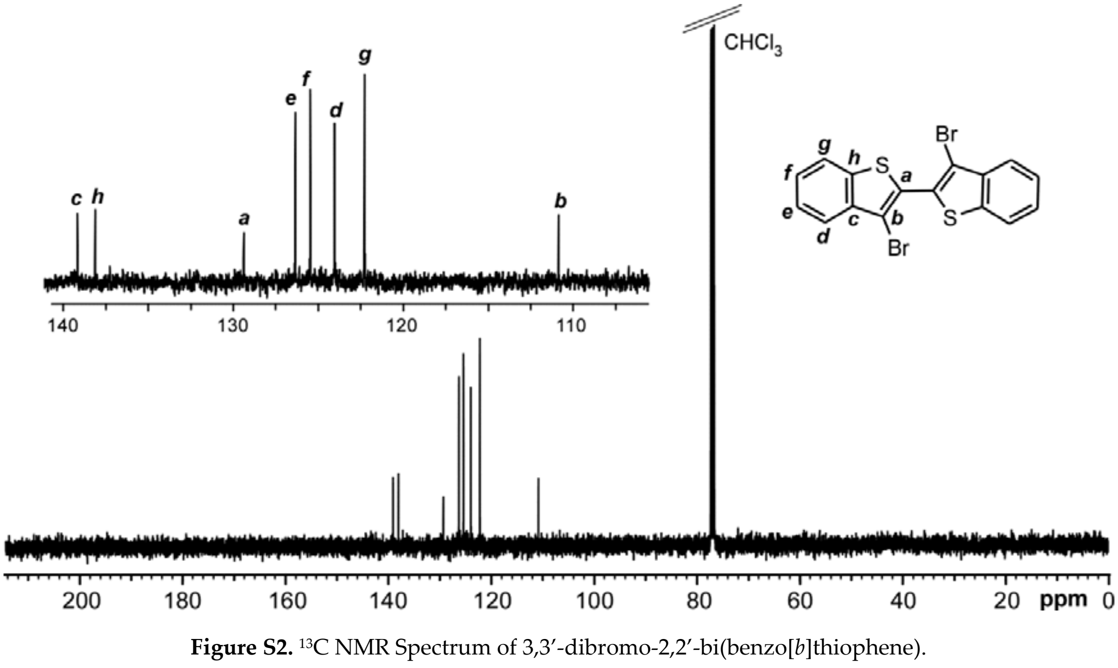

Figure S2. ^{13}C NMR Spectrum of 3,3′-dibromo-2,2′-bi(benzo[*b*]thiophene).

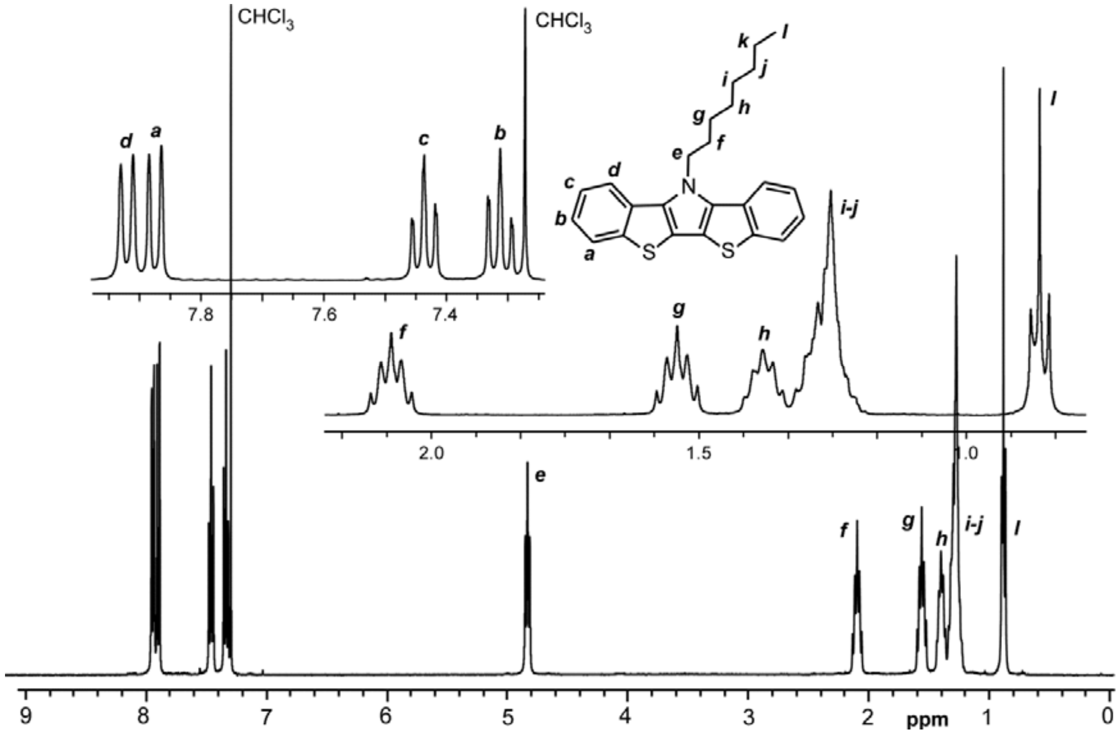

Figure S3. ^{1}H NMR Spectrum of *N*-octylbis[1]benzothieno[3,2-*b*:2',3'-*d*]pyrrole.

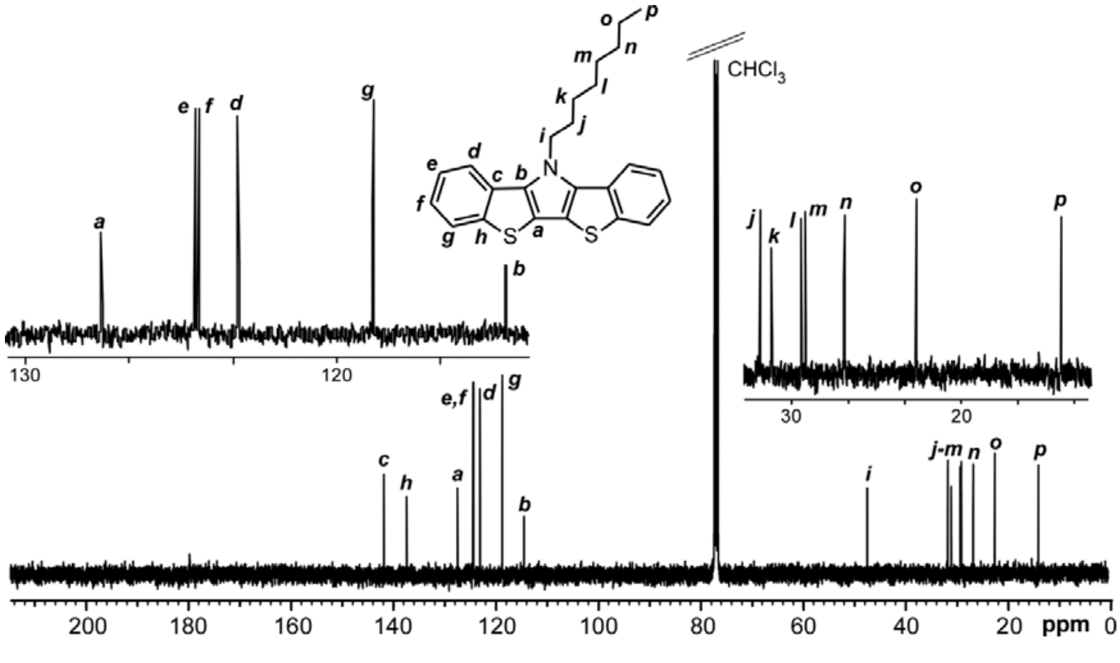

Figure S4. ^{13}C NMR Spectrum of *N*-octylbis[1]benzothieno[3,2-*b*:2',3'-*d*]pyrrole.

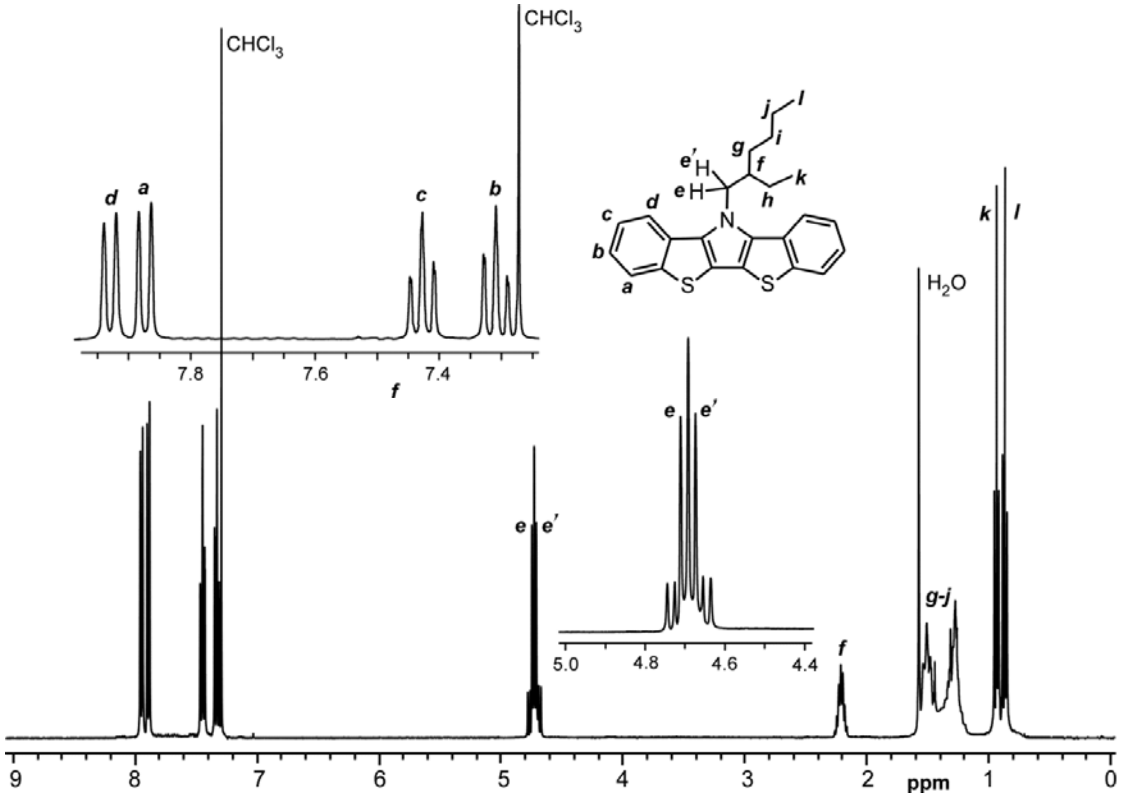

Figure S5. ^{1}H NMR Spectrum of *N*-(2-ethylhexyl)bis[1]benzothieno[3,2-*b*:2',3'-*d*]pyrrole.

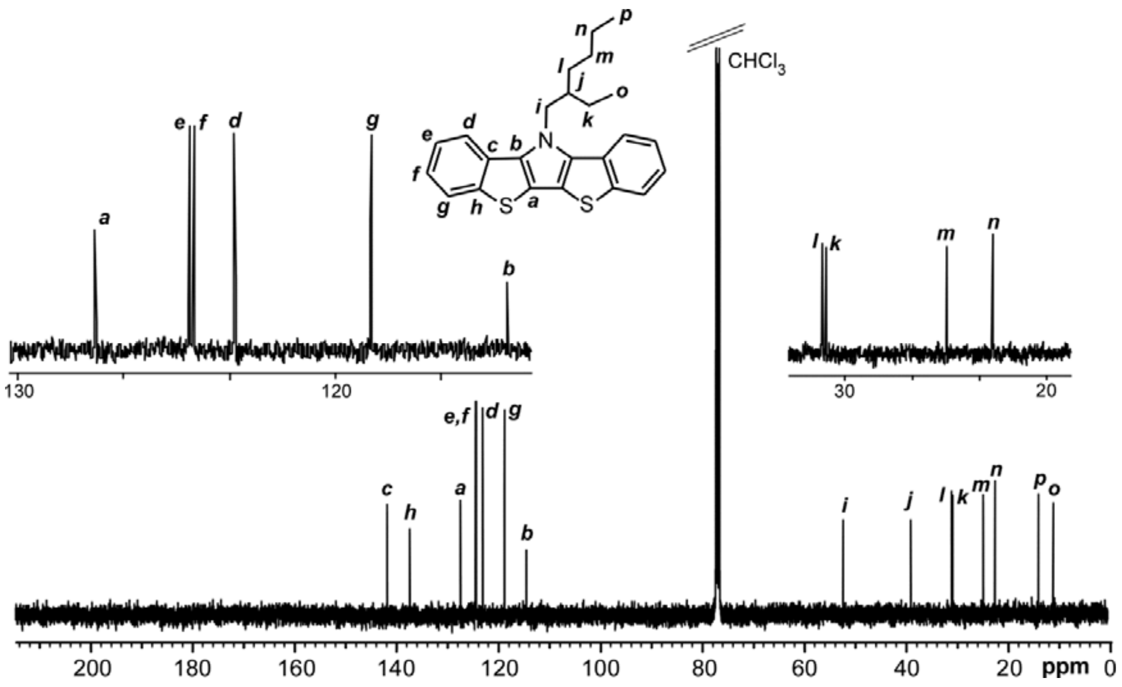

Figure S6. ^{13}C NMR Spectrum of *N*-(2-ethylhexyl)bis[1]benzothieno[3,2-*b*:2',3'-*d*]pyrrole.

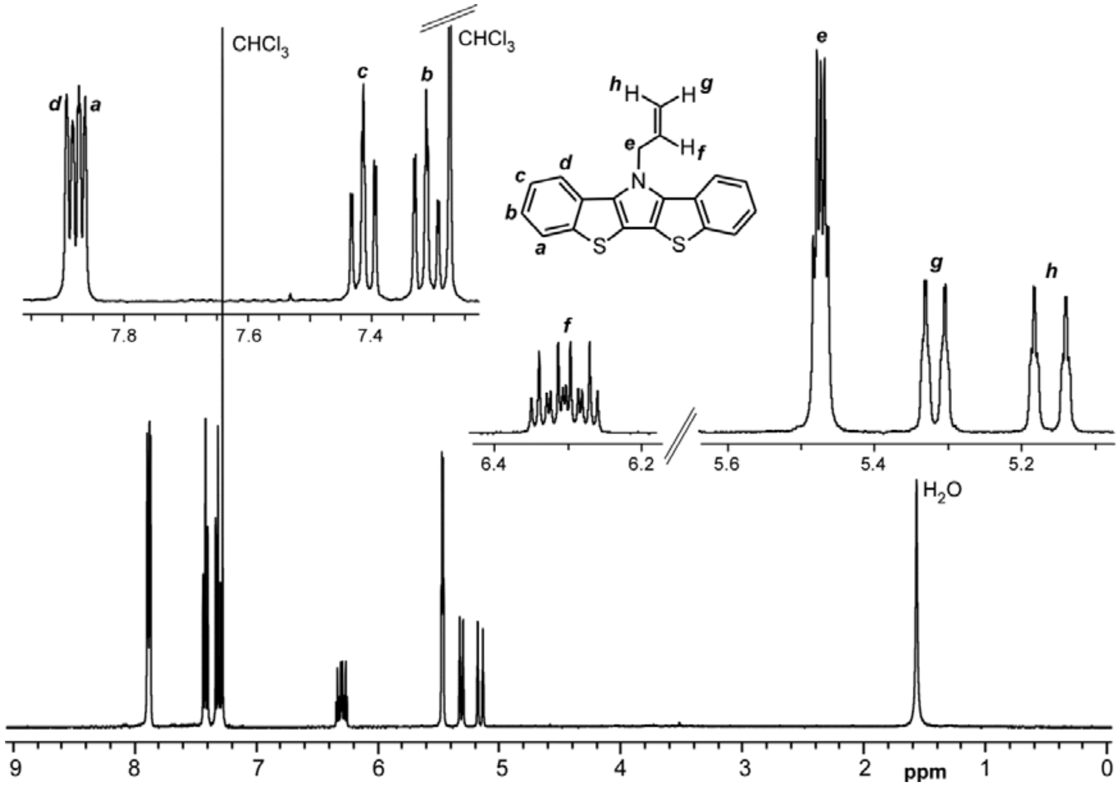

Figure S7. ^{1}H NMR Spectrum of *N*-allylbis[1]benzothieno[3,2-*b*:2',3'-*d*]pyrrole.

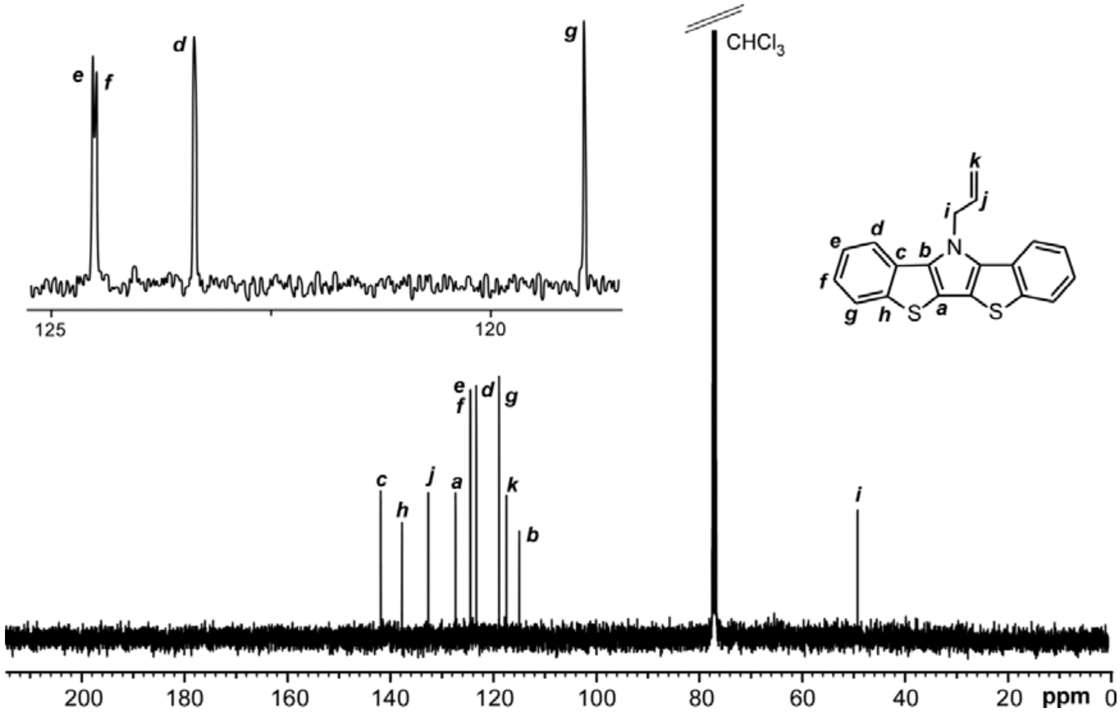

Figure S8. ^{13}C NMR Spectrum of *N*-allylbis[1]benzothieno[3,2-*b*:2',3'-*d*]pyrrole.

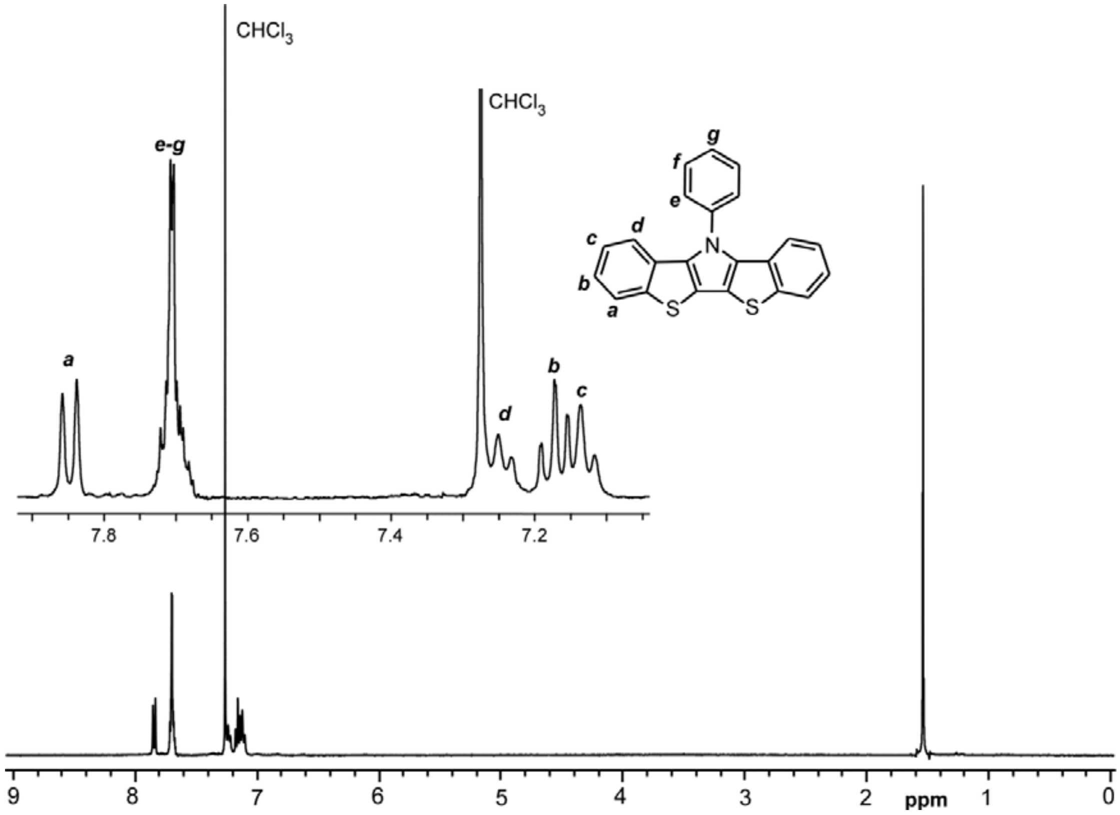

Figure S9. ^{1}H NMR Spectrum of *N*-phenylbis[1]benzothieno[3,2-*b*:2',3'-*d*]pyrrole.
